# Recovery of human gut microbiota genomes with third-generation sequencing

**DOI:** 10.1038/s41419-021-03829-y

**Published:** 2021-06-02

**Authors:** Yanfei Li, Yueling Jin, Jianming Zhang, Haoying Pan, Lan Wu, Dingsheng Liu, Jinlong Liu, Jing Hu, Junwei Shen

**Affiliations:** 1 Shanghai University of Medicine & Health Sciences affiliated Zhoupu Hospital, 201318 Shanghai, China; 2 School of Basic Medical Sciences and Shanghai Key Laboratory of Molecular Imaging, Shanghai University of Medicine and Health Sciences, 201318 Shanghai, China; 3 Shanghai OE Biotech Company, 201114 Shanghai, China; 4 Shanghai First Maternity and Infant Hospital, Tongji University School of Medicine, 201204 Shanghai, China; 5 Shanghai East Hospital, Tongji University School of Medicine, 200120 Shanghai, China

**Keywords:** DNA sequencing, Mechanisms of disease

## Abstract

Human gut microbiota modulates normal physiological functions, such as maintenance of barrier homeostasis and modulation of metabolism, as well as various chronic diseases including type 2 diabetes and gastrointestinal cancer. Despite decades of research, the composition of the gut microbiota remains poorly understood. Here, we established an effective extraction method to obtain high quality gut microbiota genomes, and analyzed them with third-generation sequencing technology. We acquired a large quantity of data from each sample and assembled large numbers of reliable contigs. With this approach, we constructed tens of completed bacterial genomes in which there were several new bacteria species. We also identified a new conditional pathogen*, Enterococcus tongjius*, which is a member of *Enterococci*. This work provided a novel and reliable approach to recover gut microbiota genomes, facilitating the discovery of new bacteria species and furthering our understanding of the microbiome that underlies human health and diseases.

## Introduction

There is increasing evidence that the gut microbiota, the human commensals, influences normal physiological functions, such as maintenance of barrier homeostasis and modulation of metabolism, inflammation, immunity, and development^1–3^. Further, disorder of the gut microbiota is a potential risk factor for various chronic diseases such as type 2 diabetes, gastrointestinal cancer, and brain disorders^4–6^. Furthermore, the gut microbiota influences drug effectiveness because of its effects on drug metabolism^7,8^. Despite decades of research, the composition of the gut microbiota remains unidentified. For example, through analysis of more than 150,000 human microbial genomes, Edoardo et al. recapitulated nearly 5000 species-level genome bins, in which 77% are not in public repositories^9^. One of the main difficulties is that many microbes are uncultivable and require isolation^9^. In addition, culture-dependent genomic research is rare in large cohorts^10^. Bypassing the need to cultivate microbes, high-throughput sequencing overcomes these shortages and is a powerful method for bacterial metagenomics.

There are two main methods for bacteria metagenome sequencing: 16S rRNA-based sequencing and whole-metagenome shotgun sequencing (WGS). The 16S rRNA-based sequencing is widely used to assess microbial communities due to its low cost, time efficiency, and ability to provide a full overview of the community^11,12^. However, as only a single region of 16s rRNA in the genome is detected, it provides limited information about the microbial community^13,14^. Furthermore, it underestimates the diversity of microbes. Compared with 16s rRNA sequencing, WGS technology examines whole bacterial genomes, and provides more accurate detection at the species and diversity levels^15^. Through WGS technology, it is feasible to assemble the entire bacterial metagenome. However, there are several drawbacks in WGS technology owing to short sequencing reads (mostly <200 bp). For example, many microbiota genomes are fragmented into numbers of contigs^16^. Besides, it is difficult to assemble the de novo genome because there is too little sequence information^16,17^. Moreover, great phenotypic differences exist even between highly related strains of the same species^18,19^, but the differences between these strains are difficult to distinguish by WGS. Therefore, there is a dire need to detect the bacterial metagenome through more powerful sequencing methods.

Third-generation sequencing (TGS) technology, also known as long-read sequencing, detects the isolated genomic DNA without amplification and produces surprising long reads (average 10–20 kb)^20^. Compared with the second-generation sequencing technology of 16s rRNA and WGS, it can detect much longer fragments, so TGS produces genome assemblies of unprecedented quality^20^. For example, Hui et al. de novo assembled a chromosome-level reference genome of the red-spotted grouper with TGS^21^. Though TGS has been used in eukaryotic genome detection extensively, but unfortunately its application in microbiota genomes have not been of sufficient scale. For instance, Johanna et al. assembled a certain specific microbial species living in the vagina with an abundance of more than 75%^22^. Thidathip et al. compared the taxonomic abundance of gut microbiota of head and neck cancer patients; while the mean length is approximately 1 kb, which is only a tenth of that of the buffalo genome (11.5 kb)^23,24^. One difficulty in the detection of gut microbiota genomes is that it is challenging to obtain high quality genomic DNA, because of complex fecal features. Therefore, large numbers of bacterial species in the gut microbiota have not yet been identified. In this study, we successfully extracted high quality gut microbial genomic DNA. By detecting the gut microbiota metagenome via Single Molecule Real-Time (SMRT) Sequencing of Pacbio, we assembled the bacteria genomes efficiently and discovered new species of bacteria.

## Materials and methods

### Sample collection and bacterial genomic DNA extraction

All methods in this study were approved by the Research Medical Ethics Committee of Shanghai University of Medicine & Health Sciences affiliated Zhoupu Hospital. Fecal samples were collected from a 34-year-old male and a 10-month-old baby and stored in an ultra-low temperature freezer (Haier, Qingdao, China). Genomic DNA extraction was performed with a FastDNA Spin Kit for Feces (MP Biomedicals, Irvine, CA, USA). To acquire sufficient high-quality gut microbiota genomic DNA, we improved the experimental procedure as follows: we added 500 mg feces in a 2 mL Lysing Matrix E tube, mixed the feces with 825 μL sodium phosphate buffer and 275 μL PLS solution, and shook the mix and vibrated for 15 s. Then, we centrifuged the samples at 14,000 × *g* for 5 min at room temperature and decanted the supernatant. Subsequently, we added 978 μL sodium phosphate buffer, shook the mix, and vibrated the mixture for 15 s, then added 122 μL MT Buffer and shook up and down gently for 5 min. Then, we placed the samples in the shaker at 4 °C for 30 min followed by centrifugation at 14,000 × *g* for 5 min and transferred the supernatant to a clean EP tube. Then, 250 μL of PPS solution was added and the samples were vigorously mixed and incubated at 4 °C for 10 min followed by centrifugation at 14,000 × *g* for 2 min. The supernatant was transferred to the binding matrix solution in a 15 mL conical tube and shook gently for 5 min. Then, the samples were centrifuged at 14,000 × *g* for 2 min and the supernatant was decanted. Afterwards, we washed the binding mixture pellet with 1 mL wash buffer #1 and transferred the binding mixture to a SPIN filter tube and centrifuged at 14,000 × *g* for 1 min. We emptied the catch tube and added 500 μL of prepared wash buffer #2 to the SPIN filter tube and gently resuspended the pellet. Afterwards, we centrifuged the samples at 14,000 × *g* twice to extract residual ethanol. Finally, we transferred the SPIN filter bucket to a clean 1.9 mL catch tube and added 100 μL TES to resuspend the genomic DNA. DNA was detected using agarose gel electrophoresis, and the top bands were isolated and purified with a DNA Purification Kit (Finegene, Shanghai, China). The DNA concentration and integrity were assessed using a NanoDrop2000 spectrophotometer (Thermo Fisher Scientific, Waltham, MA, USA).

### Library construction

For PacBio sequencing library preparation and SMRT sequencing, DNA was fragmented by a Covaris g-TUBE device (10 kb) and was concentrated with AMPure PB beads according to the manufacturer’s instructions (Beckman Coulter Co., Brea, CA, USA). DNA damage and ends were repaired in a LoBind microcentrifuge tube. Blunt ligation reaction was performed by adding 1 μL of blunt adapter (20 μM) and 1 μL of ligase to 30 μL DNA followed by incubation at room temperature for 15 min. SMRTbell™ templates were purified with AMPure PB beads and the concentration was measured by Qubit. Sequencing was performed on a PacBio Sequel instrument by OE Biotech Co., Ltd. (Shanghai, China).

### Bioinformatics analysis

Metagenome assembly was performed with flye software after obtaining valid reads. Open reading frame prediction of assembled scaffolds using prodigal was performed and translated into amino acid sequences. Non-redundant gene sets were built for all predicted genes using CD-HIT. The clustering parameters were 95% identity and 90% coverage. The longest gene was selected as the representative sequence of each gene set. The gene set representative sequence (amino acid sequence) was annotated with NR, KEGG, COG, SWISSPROT, and GO databases with an e-value of 1e^−5^. The taxonomy of the species was obtained as a result of the corresponding taxonomy database of the NR library.

### NCBI prokaryote genome databases

**Fig. 1:**
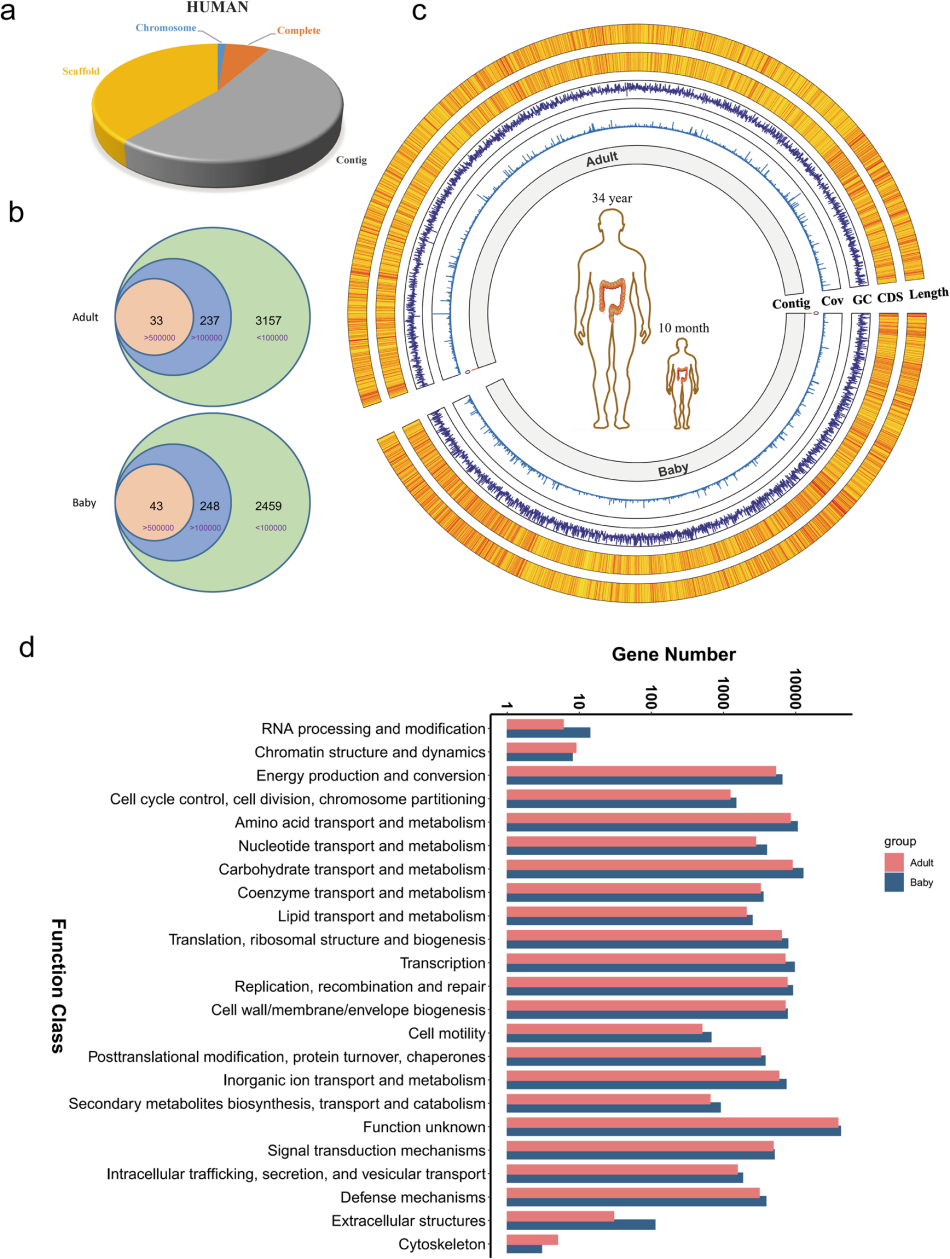
The characteristics of the two samples. **a** Pie chart comprising four classes of human bacteria sequence data in the NCBI databases: chromosome, complete, scaffold, and Contig. **b** Numbers of contigs with different lengthes acquired from the two samples, relative to the adult sample (up) and the baby sample (down). **c** The whole characteristics of the two samples. The corresponding contig length and CDS number were depicted in the first outer layer and the second outer layer, respectively; the GC content was depicted in the third outer layer; the contig number and the coverage were depicted in the first inner layer and the second inner layer, respectively. **d** Function analyses of all the CDSs of the two samples.

Prokaryotic genome data were acquired from databases in NCBI (https://www.ncbi.nlm.nih.gov/genome/browse#!/prokaryotes/). A total of 266,319 prokaryote genomes have been identified until now [chromosome (3186), complete (19,702), contig (141,127), scaffold (102,304)] (Supplementary Fig. 1). A total of 108,506 prokaryote genomes were associated with humans [chromosome (1539), complete (8179), contig (57,034), scaffold (41,754)] (Fig. 1a).

### NCBI blast

We acquired the full-length 16s rRNAs from the assembled contigs and blasted the 16s rRNAs using the NCBI database (https://blast.ncbi.nlm.nih.gov/Blast.cgi?PROGRAM=blastn&PAGE_TYPE=BlastSearch&LINK_LOC=blasthome). Then, we screened similar sequences in the order of identity, and downloaded the relative sequences. We identified the bacterial species of the assembled contigs and analyzed their evolutionary relationship.

### ClustalW and phylogenetic tree analysis

To examine the differences between full-length 16s rRNAs, we compared the 16s rRNAs and visualized the differences using BioEdit software (Borland Software Corporation, Scotts Valley, CA, USA). For phylogenetic tree analysis, we blasted the full-length 16s rRNAs in the NCBI database (https://blast.ncbi.nlm.nih.gov/Blast.cgi?PROGRAM=blastn&PAGE_TYPE=BlastSearch&LINK_LOC=blasthome) and selected the most related full-length 16s rRNAs by identity. With these 16s rRNAs, we analyzed the phylogenetic tree with Mega7^25^. The average nucleotide identity (ANI) were used to analyze whether two genomes belong to the same species^26^.

### Single bacteria analysis

Single bacteria analyses included gene prediction, non-coding RNA (ncRNA) prediction, repeat sequence prediction, nonredundant analysis, and common function potential analyses. We performed gene prediction with the Prokaryotic Dynamic Programming Genefinding Algorithm [prodigal (v2.6.3)]^27^; the results included gene number, average gene length (bp), and GC% (gene region). The ncRNA predictions were harnessed using tRNAscan-SE (v1.3.1)^28^ (tRNA), RNAmmer (v1.2)^29^ (rRNA), and Rfam(v10.0)^30^ (sRNA). Repeat sequence prediction was analyzed with RepeatMasker (v4.0.7)^31^. Common function potential analyses were performed using the Non-Redundant (https://www.ncbi.nlm.nih.gov), Swissprot (http://www.uniprot.org), KEGG (http://www.genome.jp/kegg/pathway.htmL), Cluster of Orthologous Groups of proteins (https://www.ncbi.nlm.nih.gov/COG/), comprehensive antibiotic resistance database (CARD) (https://card.mcmaster.ca)^32^, and carbohydrate-active enzymes (http://www.cazy.org) databases^33^.

### Statistical analysis

R programming language version 3.4.3 was used for statistical analysis. Statistical significance between two groups was determined using an unpaired two-tailed Student’s *t*-test. Data are presented as mean ± SD (standard deviation) or mean ± SEM (standard error of the mean) as indicated in the figure legends. *P*-values were considered statistically significant at *P* < 0.05.

## Results

### The characteristics of gut microbiota genomes

We analyzed the data of prokaryotes genomes in the NCBI database and found that complete genomes account for only a small portion (https://www.ncbi.nlm.nih.gov/genome/browse#!/prokaryotes/) (Fig. 1a and Supplementary Fig. 1a), consistent with previous research^9^. To assemble de novo complete genomes of gut microbes, we detected the gut microbiota metagenome using TGS technology. To obtain high quality gut microbiota genome samples for TGS, we attempted to improve the integrity and quantity of genome DNA. Initially, the isolated genomic DNA was short. After trying many different kits, we acquired fairly complete bacteria genomes. Then, we tried to improve the yield of genomic DNA. We isolated and purified the gut microbiota genome DNA with a DNA purification kit. Finally, we acquired two high quality samples: a 34-year-old male and a 10-month-old baby.

We detected the whole metagenome of the two samples using TGS technology. We acquired 91.7 and 81.7 Gb data from the baby and adult sample, respectively. Through analysis of genome databases in NCBI, we found that most of the bacterial genomes are larger than 0.5 Mb and the smallest bacteria genome was approximately 0.1 Mb (Supplementary Table 1 and Supplementary Fig. 1b). Therefore, we chose 0.1 and 0.5 Mb as cutoff values to analyze the assembly contigs (Fig. 1b). In the baby sample, there were 43 contigs larger than 0.5 Mb and 248 contigs larger than 0.1 Mb. Similarly, in the adult sample, there were 33 contigs larger than 0.5 Mb and 237 contigs larger than 0.1 Mb (Fig. 1b). We analyzed the 16s rRNA sequences from the longest contig (Contig_511). The six 16S rRNA sequences of contig 511 were almost the same, with only two different bases in the over 1.5 kb sequence (Supplementary Fig. 1c). Interestingly, the 16S sequence in contig_511 had fewer differences than that in *Escherichia coli* (U00096.3) (Supplementary Fig. 1c). This result suggested that the methods we used were reliable (Supplementary Fig. 1c). Moreover, the contig length was significantly related to the number of coding sequences (CDSs) (Fig. 1c). Through analysis of the functional potential of CDSs, we found that many CDSs were correlated with the metabolism of amino acids and carbohydrates. Interestingly, the functional potential of a large number of genes was unknown, indicating that there are still numerous valuable genes worth investigating in gut microbes (Fig. 1d).

### Aspects of the contigs in the two samples

**Fig. 2:**
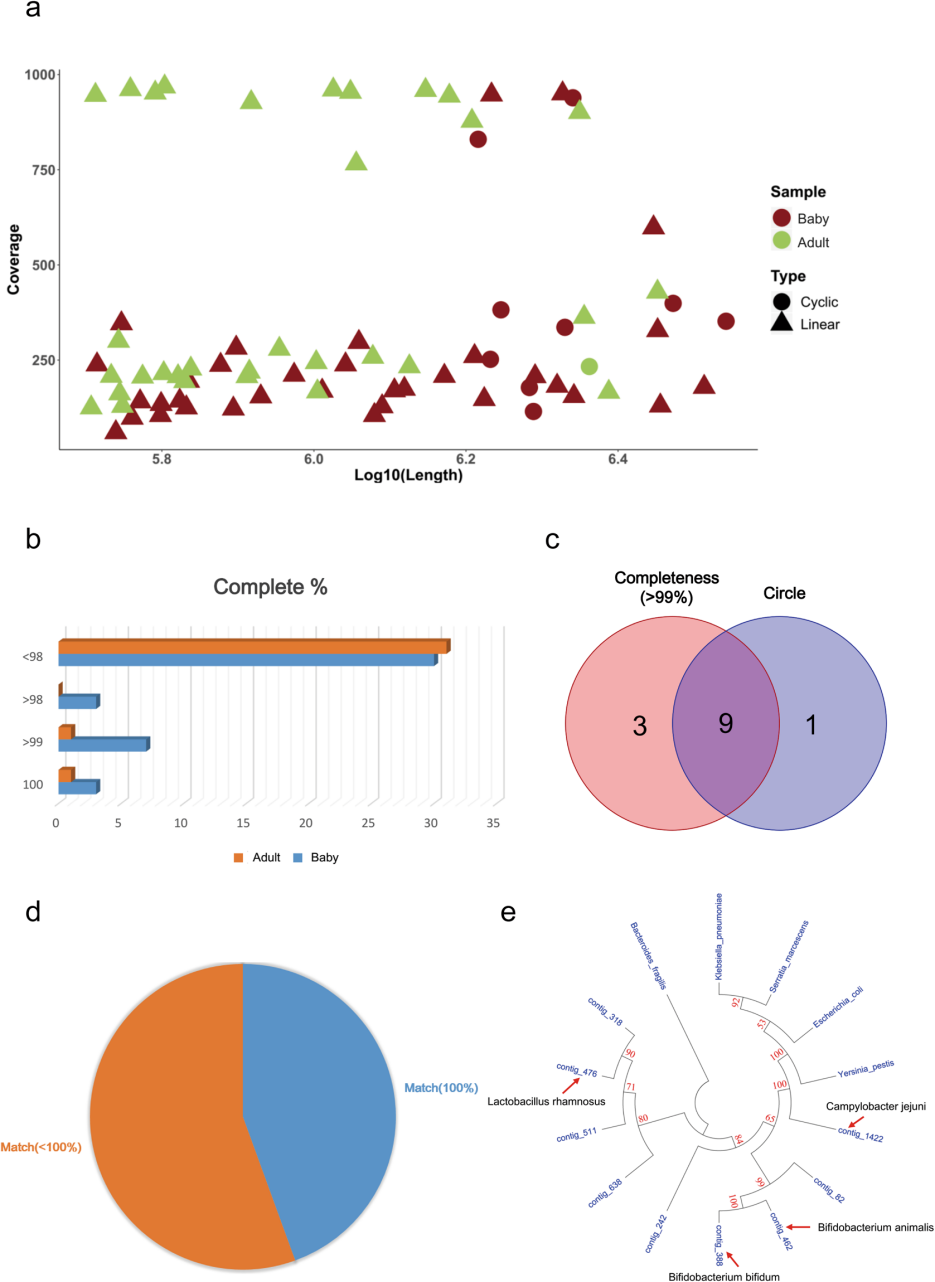
The different aspects of the contigs (longer than 0.5 Mb) in the two samples. **a** The length and coverage of the contigs in the two samples (**longer than** 0.5 Mb). **b** Complete analysis of the contigs in the two samples. **c** Wenn diagram shows the number of contigs (Complete ≥99% or circle). **d** Pie diagram shows the number of the nine genomes whether they match public genomes. **e** Phylogenetic tree of the nine assembled genomes, each arrow shows the completely matched contig.

Because most bacterial genomes are greater than 0.5 Mb, we further analyzed the contigs greater than 0.5 Mb in the two samples (Supplementary Table 2). Among these contigs, 10 were circular (Fig. 2a) and 12 were more than 99% complete (Fig. 2b). Nine contigs were both circular and complete (>99%) (Fig. 2c). Therefore, we focused on these nine contigs and analyzed their features. The full-length 16S rRNA blast analyses showed that less than 50% (four of nine contigs) of these contigs could be completely matched with existing sequences in the NCBI databases (Fig. 2d, e; indicated by red arrow). Five were not completely matched, indicating they might be new species or subspecies (Fig. 2d, e). To investigate the taxonomic location of the nine contigs, we analyzed them by phylogenetic analysis using full-length 16S rRNA. By comparison with common bacteria such as *Bacterioides fragilis* and *E*. *coli*, we successfully identified their taxonomic location in the phylogenetic tree (Fig. 2e).

### Genomic characteristics of the five non-matched genomes

**Fig. 3:**
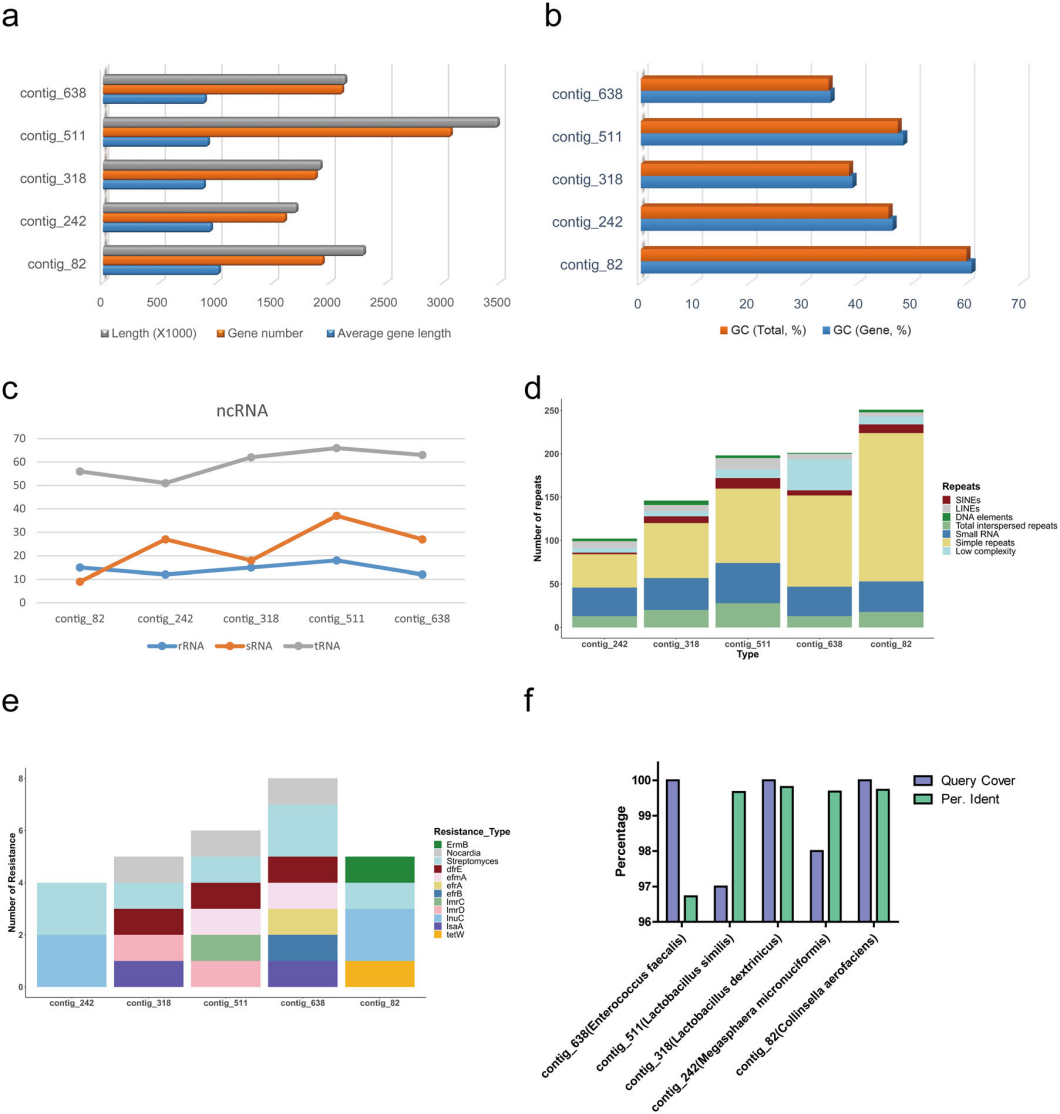
The genome analysis of the five non-match genomes. **a** The genome length and gene number of the five non-match genomes. **b** The GC content(total and gene) of the contigs in the two samples of the five genomes. **c** The ncRNA analysis (rRNA, tRNA and sRNA) of the five genomes. **d** The repeat sequences of the five genomes. **e** The antibiotic potential of the five genomes. **f** The blasted result of the full-length 16S rRNAs of the five sequences in the NCBI database. The reference bacteria are shown in parentheses.

We further analyzed the five genomes that could not be completely matched. Their genome lengths ranged from 1.5 to 3.5 Mb (Supplementary Table 3 and Fig. 3a). Despite the large difference in genome length, the average length of each gene was similar (Fig. 3a). In contrast, the difference in GC content between these genomes was large (Fig. 3b). We also analyzed some special structures of the genomes, including ncRNA and repeat sequences. Interestingly, the number of rRNAs and tRNAs in each genome was correlated (Fig. 3c). Each genome had all types of repeat sequences; simple repeats were the major repeat type (Fig. 3d). Intriguingly, we found that all five bacteria exhibited streptomycin resistance, possibly a result of long-term antibiotic use (Fig. 3e) and suggesting that the extensive use of antibiotics may have irreversibly transformed the human gut microbiome. To determine the classification to which they belong, we blasted the full-length 16S rRNAs in NCBI databases (https://blast.ncbi.nlm.nih.gov/Blast.cgi?PROGRAM=blastn&PAGE_TYPE=BlastSearch&LINK_LOC=blasthome). Four contigs (contig_82, contig_242, contig_318, and contig_511) had high identities (>99%), while the identity of contig_638 was less than 97% (Fig. 3f).

### Phylogeny of four high identity non-matched genomes

**Fig. 4:**
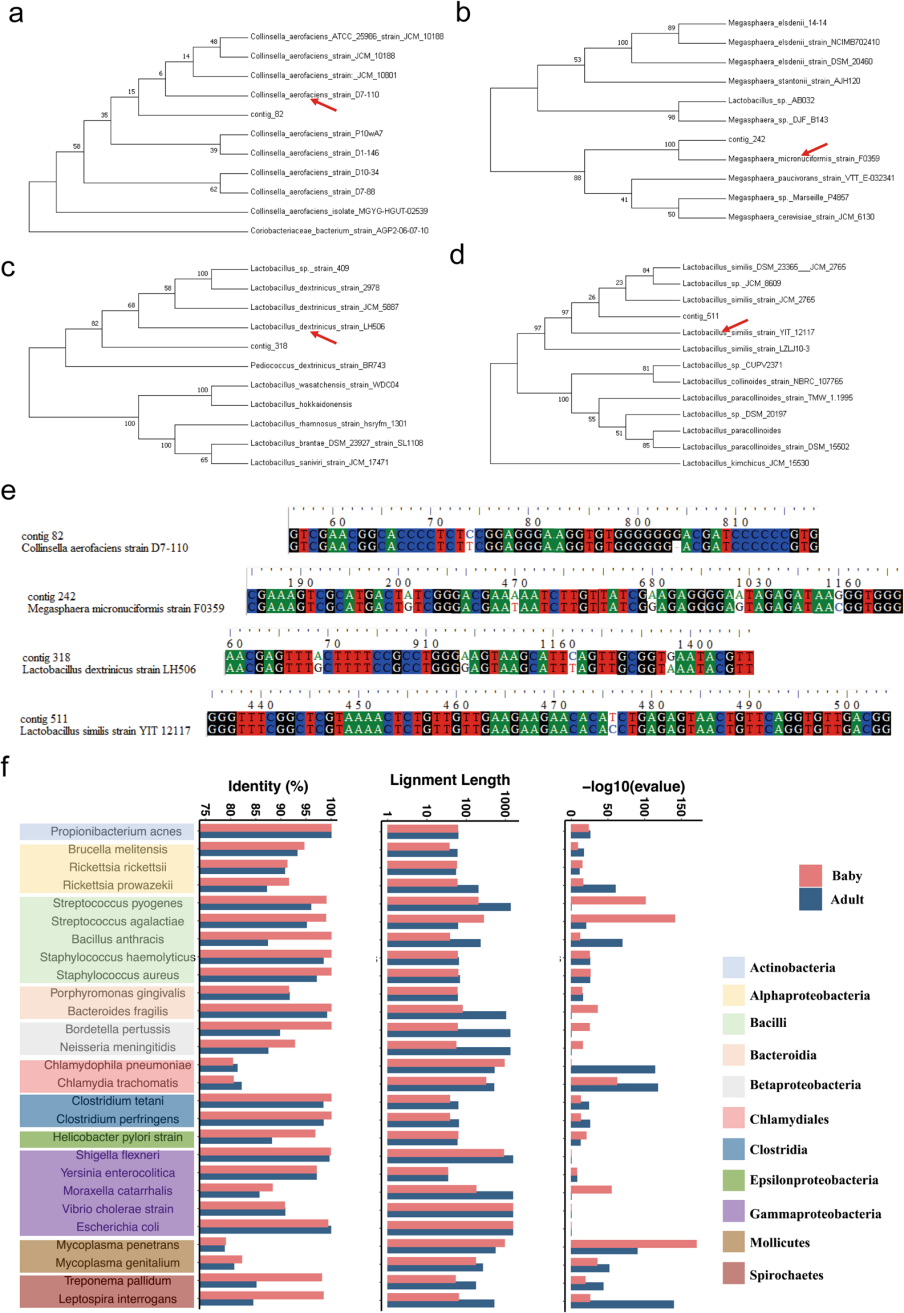
Phylogeny of four high identity non-match genomes. **a**–**d** Phylogenetic tree of the high identity non-match genomes through full-length 16s rRNAs, showing their accurate taxonomic location. **e** ClustalW multiple alignment of the full-length 16s rRNAs of these genomes with that of the related bacterial strains. **f** Conditional pathogens Shigella flexneri lived in the gut of adult and baby.

We then analyzed the bacteria with identities of more than 99%, and detected their accurate taxonomic location by phylogenetic tree analysis. We blasted the full-length 16s rRNAs of these genomes with the NCBI databases, and selected the most related species (rank by identity) for further analysis. Contig_82 belongs to *Collinsella erofaciens* and might be a new subspecies (Fig. 4a). Similarly, contig_242 is a subspecies of *Megasphaera micronuciforrnis*, contig_318 is a subspecies of *Lactobacillus dextrinicus*, and contig_511 is a subspecies of *Lactobacillus smilis* (Fig. 4b–d). Analysis of non-redundant database annotations confirmed these results (Supplementary Fig. 3a–d). We then compared the full-length 16s rRNAs of these genomes with those of related bacterial strains, and found that the differences between them were small (Fig. 4e). The most considerable difference in contig_318 was only five bases among more than 1500 bases (Fig. 4e). Interestingly, *Megasphaera micronuciforrnis* (contig_242) was a conditional pathogen; therefore we sought to evaluate whether healthy people also carry other conditional or common pathogens. We selected 27 common and conditional pathogens, including *E*. *coli*, *Staphylococcus aureus*, *Shigella flexneri*, and *Helicobacter pylori*. Only *S*. *flexneri* was found in the gut of the adult and baby samples, while other pathogenic bacteria were not found (Fig. 4f).

### Genomic aspects of the new species *Enterococcus tongjius*

**Fig. 5:**
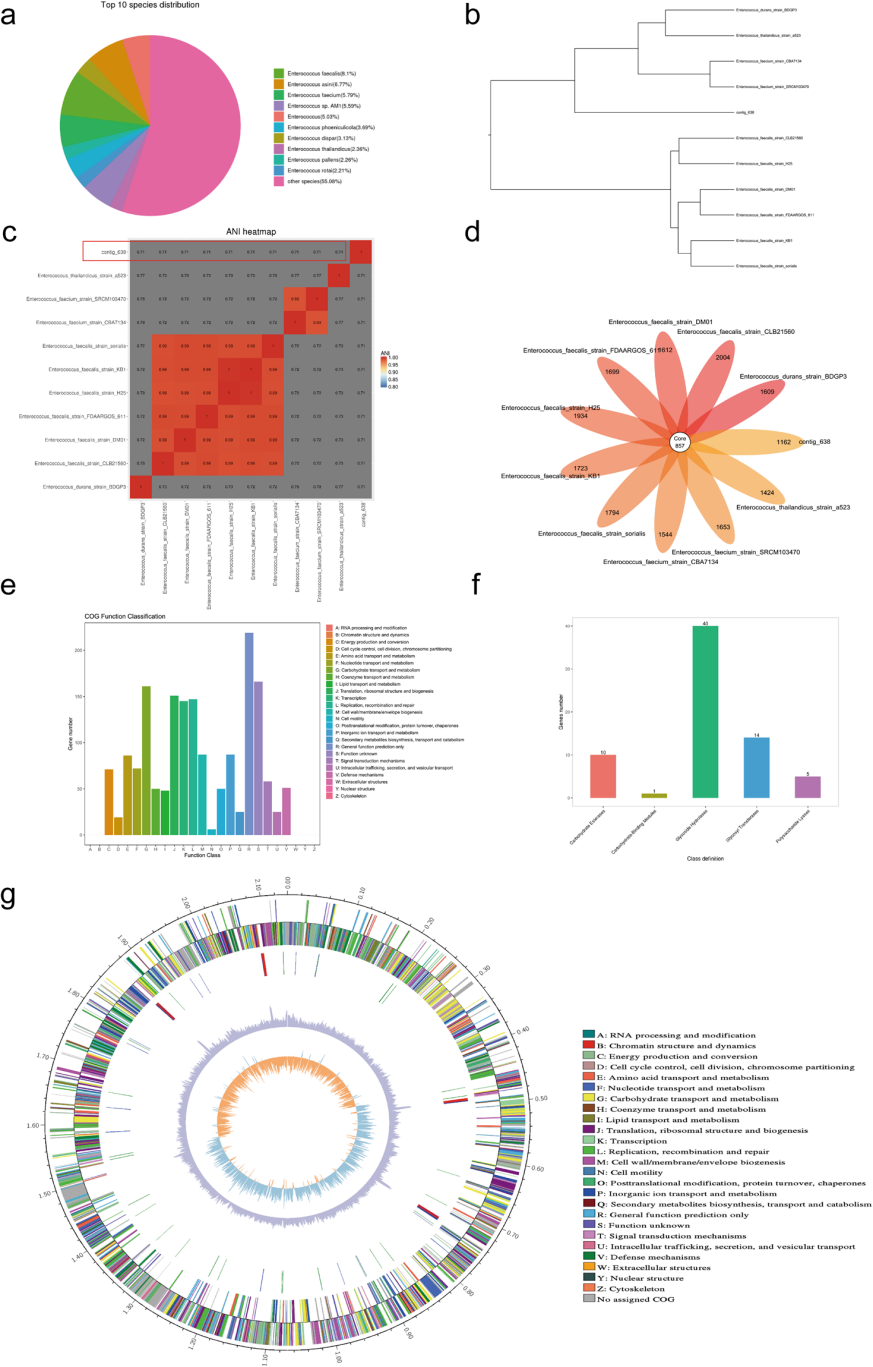
Genome aspects of the new species of *Enterococcus tongjius*. **a** Non-redundant database annotations of contig_638. **b** Phylogenetic tree of the high contig_638 through whole genomes. **c** The ANI analyses showed that contig_638 was a total new species since the ANI score was only 0.71, much lower than 0.9. The red frame displayed the scores of contig_638. **d** A flower-plot schematic representation illustrates the number of predicted core (857) genes and distinctive genes**. e** Function analyses of the CDSs of the *Enterococcus tongjius*. **f** Carbohydrate-active enzymes analysis of the *Enterococcus tongjius*. **g** Circos diagram of the whole genome of the *Enterococcus tongjius* (From the inner to the outer are GC skew, GC content, non-coding RNA (rRNA red, tRNA blue, sRNA green), lagging strand COG annotation, leading strand COG annotation).

We analyzed contig_638, which showed poor identity. Through the analysis of non-redundant database annotations, we found it difficult to find a bacterium to match contig_638, because the most similar *Enterococcus faecalis* showed only 8% similarity (Fig. 5a). We sought to determine its classification through NCBI blast and 16s rRNA phylogenetic tree analysis. Excitingly, we discovered that this bacterium belongs to Enterococcus (Supplementary Fig. 4a). Compared with other similar bacteria *Enterococcus saccharolyticus* and *Enterococcus faecalis*, we found that there were considerable differences in the 16s rRNA sequences (Supplementary Fig. 4b). To confirm whether *Enterococcus tongjius* is a new species, we compared its genome with some similar *Enterococci* genomes (Fig. 5b). The ANI results showed that it was a total new species since the ANI score was much lower than 0.9 (Fig. 5c). Besides, the flower-plot showed that though *Enterococcus tongjius* shared only 867 core genes with other *Enterococci*; it had 1162 distinctive genes (Fig. 5d). Because it was found in Tongji University, we named it *Enterococcus tongjius*. We then analyzed the function potential of the genome and found that many genes might be correlated with carbohydrate and lipid metabolism (Fig. 5e). Interestingly, the function potential of many genes was not clear (Fig. 5e). Further analysis of sugar metabolism showed that most genes were associated with hydrolysis (Fig. 5f). Finally, we analyzed the whole genome of *Enterococcus tongjius* and found it contains a total of 2.1 M bases, and the GC content is relatively low (34.49%), which is also consistent with other species of *Enterococci* (Fig. 5g). It had 2111 genes, 12 rRNAs, and 63 tRNAs (Fig. 5g).

## Discussion

Extensive studies of the human gut microbiota have been reported in past decades. However, large numbers of species in the gut microbiota remain unknown. By improving the extraction method, we successfully acquired high-quality genomic DNA of the gut microbiota, and obtained approximately 85 Gb TGS data from each sample, which was much greater than previous studies^23,34,35^. We successfully assembled nine bacterial genomes and large numbers of contigs using this method. Interestingly, more than 50% of the genomes (five of nine bacterial genomes) might be new human species or subspecies. Using large-scale metagenomic assembling, Edoardo et al. and Alexandre et al. uncovered thousands of new species of bacteria^9,36^. These results, together with our work, indicated that further investigation of gut microbiota is worthwhile. Furthermore, the approach used in this study will help assemble the genome of unknown bacteria, and thus might facilitate the Human Microbiome Project and the Genomic Encyclopedia of Bacteria and Archaea^37,38^. Finally, bacteria are widely distributed in various environments, such as natural lakes, oceans, and soils. Therefore, it is of great interest to investigate bacteria living in different environments with our methods.

Compared with the metagenome, genes in the microbial genome have traditionally been underestimated. For example, Hila et al. found that there are a fairly large number of unknown small proteins in the human microbiome^39^. Intriguingly, our work discovered more than 10,000 unknown genes without known domain (Fig. 1d). These findings indicated that a vast number of unidentified genes are needed to be explored in the human microbiome. Importantly, some bacterial genes are valuable for human health. For example, an anti-inflammatory protein from *Faecalibacterium prausnitzii* could inhibit the NF-κB pathway in intestinal epithelial cells^40^. Via KEGG analysis, we found that most genes in the two samples were closely associated with human basic physiological activities (Supplementary Fig. 3a, b). In addition, bacterial genes are potential disease diagnostic indicators because they can pass through intestinal epithelial cells and enter the plasma^41^. The exploration of drug-metabolizing enzymes from the gut microbiota may be useful in drug development and personalized medicine^7,42^. Our research will help discover bacterial proteins linking human health and diseases.

In the nine assemble genomes, we discovered a new bacterium, *Enterococcus tongjium*. We discovered that it belongs to *Enterococcus*, a ubiquitous Gram-positive genus with low-GC genomes. Many members of this genus are pathogenic bacteria or conditional pathogens, because of their role as primary causative agents of healthcare-associated infections^43,44^. Therefore, this bacterium might be a conditional pathogen. We also found two other conditional pathogens: *Megasphaera micronuciforrnis* and *Shigella flexneri*. An important question remains: what matters to human health: the quality or quantity of microbes^45^? The results in this work suggested that the quality and quantity of microbes were both important for our health.

Despite the promise that this study holds for gut microbiota, it is important to note its limitations. First, the lengths of bacterial genomes acquired were between 1.5 and 3.5 Mb, much smaller than that of *E*. *coli* str. K-12 (4.64 Mb). We found that the contig lengths were closely correlated with the coverage (Supplementary Fig. 5). Therefore, it is essential to improve the data quantity of each sample to obtain longer contigs. With the continuous development of TGS technology, it is probable that more sequencing data can be acquired in a single sample. Second, although we established an effective method, it is still not easy to acquire sufficient high quality genomic DNA, and we only harvested two samples. It is urgent to improve these methods and to analyze more human fecal samples. At last, we also tried to cultivate the newly identified species, *Enterococcus tongjius*, with multiple traditional microbiological means, including different medium and antibiotics; but we have not isolated *Enterococcus tongjius* clone successfully until now. Our inability to culture this bacterium has limited our understanding of its ecological role in the intestinal environment and its relation to human’s health.

In summary, we established an effective extraction method to obtain high quality gut microbiota genomic DNA and detected the samples with TGS technology. We identified not only a large number of unknown genes, but also several new subspecies and species with our methods. This work provided a novel and reliable framework for exploring gut microbiota genomes, improving our understanding of the mechanisms that underlie the role of the microbiome in health and disease.

## Supplementary information

Supplementary legends

Supplemental Fig. 1

Supplemental Fig. 2

Supplemental Fig. 3

Supplemental Fig. 4

Supplemental Fig. 5

Supplementary Tab. 1

Supplementary Tab. 2

Supplementary Tab. 3

## Data Availability

The raw sequencing data generated from this study have been deposited in NCBI SRA (https://www.ncbi.nlm.nih.gov/sra) under the accession number PRJNA717332.
