# Supplementary material for: Recovery of human gut microbiota genomes with third-generation sequencing
Source: Cell Death Dis. 2021 Jun 2;12(6):569. doi: 10.1038/s41419-021-03829-y (PMC8172872; doi:10.1038/s41419-021-03829-y)
Supplement: Supplementary file 1 — Supplementary legends [file 41419_2021_3829_MOESM1_ESM.docx]

**Supplemental material**

**Supplemental Fig. 1 | The characters of prokaryote sequence in the NCBI databases. a,** Pie chart shows 4 classes of prokaryote sequence data in the NCBI dataset: Chromosome, Complete, Scaffold and Contig. **b,** Genome length and the GC content of human bacteria sequence data in the NCBI databases. The red line: 0.5 Mb **c,** ClustalW multiple alignment of the full-length 16s rRNAs of contig_511 and E. coli (U00096.3).

**Supplemental Fig. 2 | Non-redundant database annotations of four high identity non-match genomes. a,** Non-redundant database annotations of Contig_82. **b,** Non-redundant database annotations of Contig_242. **c,** Non-redundant database annotations of Contig_318. **d**, Non-redundant database annotations of Contig_511.

**Supplemental Fig. 3 | KEGG classification of the all the CDSs of the two samples. a,** KEGG classification of the all the CDSs of the baby sample. **b,** KEGG classification of the all the CDSs of the adult sample.

**Supplemental Fig. 4 | Phylogeny of contig_638 with high identity genomes. a,** Phylogenetic tree of the high contig_638 through full-length 16s rRNAs**. b,** ClustalW multiple alignment of the full-length 16s rRNAs of *Enterococcus tongjius* with that of the related bacterial strains**.**

**Supplemental Fig. 5 | The lengths of the contigs are correlated to the coverage.**

**Supplementary Tab. 1 | The characters of prokaryote sequence in the two samples**.

**Supplementary Tab. 2 | The aspects of long sequences (>0.5 Mb) in the two samples.**

**Supplementary Tab. 3 | The genome analysis of the five non-match genomes.**
